# Comparative analysis of prognostic scoring systems in predicting severity and outcomes of Omicron variant COVID-19 pneumonia

**DOI:** 10.3389/fmed.2024.1419690

**Published:** 2024-06-18

**Authors:** Ruiqin Ni, Mingmei Zhong, Mengrong Xie, Zhen Ding

**Affiliations:** ^1^Graduate School, Bengbu Medical University, Bengbu, China; ^2^Third Affiliated Hospital of Anhui Medical University, Hefei, China; ^3^Wannan Medical College, Wuhu, China

**Keywords:** Omicron COVID-19 pneumonia, disease severity, prognosis, clinical classification, scoring systems

## Abstract

**Background:**

The global spread of Coronavirus Disease 2019 (COVID-19) underscores the urgent need for reliable methods to forecast the disease’s severity and outcome, thereby facilitating timely interventions and reducing mortality rates. This study focuses on evaluating the clinical and laboratory profiles of patients with Omicron variant-induced COVID-19 pneumonia and assessing the efficacy of various scoring systems in prognosticating disease severity and mortality.

**Methods:**

In this retrospective analysis, we examined the clinical records of 409 individuals diagnosed with Omicron variant COVID-19 pneumonia. We documented the Pneumonia Severity Index, CURB-65, and MuLBSTA scores within the first 24 h and analyzed the sensitivity, specificity, positive predictive value, negative predictive value, and the area under the receiver operating characteristic curve for each scoring system to ascertain their predictive accuracy for disease severity and fatality risk.

**Results:**

The cohort’s median age was 78 years, predominantly presenting with fever, cough, expectoration, fatigue, and gastrointestinal symptoms. Factors such as expectoration, fatigue, Glasgow Coma Scale score, lactate dehydrogenase levels, procalcitonin, creatinine levels, and co-occurrence of acute respiratory distress syndrome were identified as independent predictors of disease severity. Furthermore, age, oxygenation index, glucose levels, lactate dehydrogenase, and septic shock were independently associated with mortality. For severe disease prediction, the CURB-65, PSI, and MuLBSTA scores demonstrated sensitivities of 65.9%, 63.8%, and 79.7%, respectively, with specificities of 63.8%, 76.8%, and 60.9%, and AUROCs of 0.707, 0.750, and 0.728. To predict mortality risk, these scores at cutoffs of 1.5, 102.5, and 12.5 exhibited sensitivities of 83.3%, 96.3%, and 70.4%, specificities of 59.4%, 60.8%, and 65.4%, and AUROCs of 0.787, 0.850, and 0.736, respectively.

**Conclusion:**

The study cohort predominantly comprised elderly individuals with pre-existing health conditions. Elevated lactate dehydrogenase emerged as a significant marker for both disease severity and prognosis, sputum production, gastrointestinal symptoms, GCS score, creatinine, PCT, and ARDS as independent predictors of disease severity, and age, oxygenation index, glucose levels, and septic shock as independent mortality predictors in COVID-19 pneumonia patients. Among the scoring systems evaluated, Pneumonia Severity Index demonstrated superior predictive capability for both disease severity and mortality, suggesting its utility in forecasting the clinical outcomes of Omicron variant COVID-19 pneumonia.

## 1 Introduction

COVID-19 remains a pervasive infectious disease worldwide. Over 2 years into the pandemic, the virus has mutated extensively, leading to a decline in overall morbidity and mortality rates. Despite this, COVID-19 continues to pose a substantial threat, particularly to older individuals and those with comorbidities who face a heightened risk of death, while younger patients are not exempt from severe outcomes. The World Health Organization (WHO) currently recognizes five variants of concern: Alpha, Beta, Gamma, Delta, and Omicron, with the latter first identified in South Africa and classified by the WHO on 26 November 2021 (1). Notably, the Omicron variant, characterized by increased transmissibility and lower mortality compared to its predecessors, has become the dominant strain globally (2).

The continuous evolution of COVID-19 presents ongoing challenges, with many aspects of the disease yet to be fully understood. The lack of robust early indicators for disease severity complicates clinical assessments, which may be influenced by subjective judgments. Thus, there is an imperative need for straightforward, objective methods to assess patient conditions, augmenting clinical discretion with quantitative data.

Initial studies during the pandemic highlighted the association between respiratory system impairment upon hospital admission and adverse outcomes (3). However, much of the extant literature focuses on the clinical manifestations of the original viral strain, with comparatively scant data on the Omicron variant. This study aims to elucidate the clinical features of Omicron-induced COVID-19 pneumonia and evaluate the efficacy of various prognostic scoring systems in predicting disease severity and patient outcomes. By identifying the most effective scoring system, this research aims to facilitate the early recognition of patients at risk of severe illness or death, thereby enabling timely interventions, optimizing survival prospects, and aiding healthcare providers in the judicious allocation of medical resources.

## 2 Data and methods

### 2.1 Study design and selection criteria

This retrospective cohort study was conducted at the Third Affiliated Hospital of Anhui Medical University, which compiled clinical data (demographics, clinical manifestations, comorbidities, laboratory findings, etc.) from patients diagnosed with COVID-19 pneumonia between 1 November 2022 and 23 January 2023.

#### 2.1.1 Selection criteria

**Figure 1:**
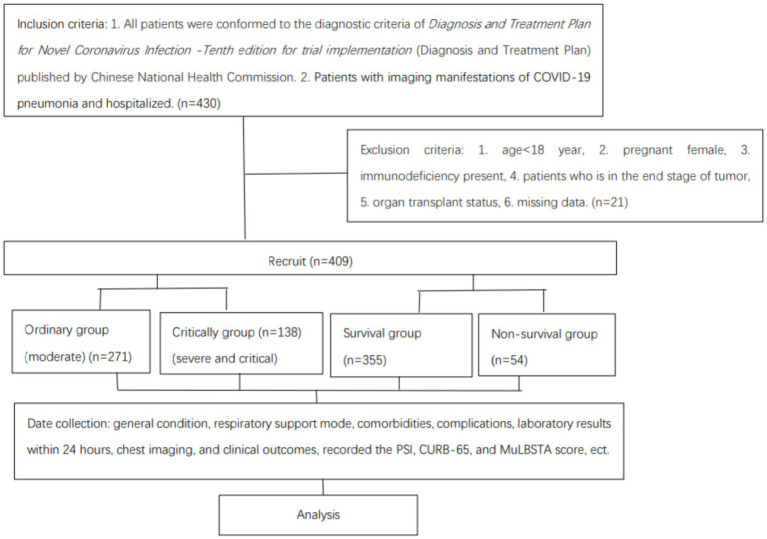
Group assignment.

Inclusion: (1) Patients meeting the diagnostic criteria outlined in the “Diagnosis and Treatment Plan for Novel Coronavirus Infection—Tenth Edition” by the Chinese National Health Commission. (2) Patients exhibiting radiological signs of COVID-19 pneumonia admitted for treatment. Exclusion: Individuals under 18 years, pregnant women, those with immunodeficiency, patients in terminal stages of cancer, individuals with organ transplants, and cases with incomplete data were excluded (Figure 1). The Clinical Research Ethics Committee of the Third Affiliated Hospital of Anhui Medical University granted ethical approval for this study (Approval No. LUN 2023 [47]).

### 2.2 Methodology

#### 2.2.1 Data acquisition

Clinical and demographic details (age, sex, symptoms including fever, cough, sputum production, fatigue, gastrointestinal symptoms, shortness of breath, oxygen saturation, chest discomfort, respiratory and heart rates, mean arterial pressure, oxygenation index), respiratory support methods (nasal cannula, oxygen mask, high-flow nasal cannula (HFNC), non-invasive and invasive ventilation), complications (acute respiratory distress syndrome (ARDS), septic shock, bacterial co-infections), existing health conditions (hypertension, cardiovascular diseases, chronic pulmonary conditions, diabetes, cerebrovascular diseases, cancer, chronic kidney diseases, etc.), and laboratory findings within 24 h of admission (white blood cell count, neutrophil and lymphocyte counts, hemoglobin levels, C-reactive protein (CRP), procalcitonin (PCT), platelet count, blood urea nitrogen (BUN), creatinine, sodium, glucose, etc.) were collected through the hospital’s electronic medical records. The study also recorded Pneumonia Severity Index (PSI), CURB-65, and MuLBSTA scores for each patient. Bacterial co-infections means that the patient who diagnosed as COVID-19 pneumonia has symptoms of cough purulent sputum, elevated hemogram in laboratory examination, detection of bacteria in microbiological examination, etc.

Patients were categorized into moderate, severe, and critical groups based on the clinical classifications in the “Diagnosis and Treatment Plan” (4). Patients were further divided into survival and non-survival groups post-hospitalization. The meaningful predictive variables in univariate analysis were included in the regression analysis, forward conditional was used to identify independent risk factors for disease severity and mortality. Evaluation of sample size by power analysis, with an alpha and effect size of 0.05 and 0.35, when the study-power was projected as 80%, in the prognosis (u = 28) the sample size at least 129, and in the severity of disease (u = 36), the sample size at least 140, the sample size of this study is 409. Receiver operating characteristic (ROC) curves were plotted to evaluate the efficacy of various scoring systems in predicting disease severity and mortality, employing the Youden index to determine optimal cutoff points, followed by calculation of sensitivity, specificity, positive predictive value (PPV), negative predictive value (NPV), and their confidence intervals.

The MuLBSTA score is a prognostic model for 90-day mortality in viral pneumonia, incorporating six criteria: multilobar infiltration, lymphopenia, bacterial co-infection, smoking history, hypertension, and age (5). The CURB-65 score assesses the severity of community-acquired pneumonia, based on confusion, uremia, respiratory rate, blood pressure, and age ≥ 65 years (6). The PSI score includes 20 variables spanning demographics, pre-existing conditions, physical examination findings, and laboratory and radiographic data (7).

### 2.3 Statistical analysis

Data were processed using SPSS software version 26. Non-parametric data are presented as median(interquartile ranges) [M(IQR)], analyzed using the Mann–Whitney U test. Categorical variables, expressed as frequencies and percentages, were compared using Pearson’s chi-squared test. Variables significant in univariate analyses (*p* < 0.05) were included in a multivariate logistic regression model to identify risk factors for disease severity and mortality. The performance of each scoring system in predicting disease outcomes was assessed via sensitivity, specificity, NPV, PPV, and area under the receiver operating characteristic curve (AUROC). Statistical significance was established at *p* < 0.05.

## 3 Results

### 3.1 Clinical characteristics

The cohort comprised 409 individuals, including 173 males and 236 females, with a median age of 78 years. Stratification of COVID-19 pneumonia cases into three categories (271 moderate, 112 severe, and 26 critical) revealed significant distinctions across age, sex, heart rate, respiratory rate, body temperature, peripheral oxygen saturation, Glasgow Coma Scale (GCS) score, existing health conditions (chronic pulmonary diseases, rheumatic immune disorders), and clinical manifestations (sputum production, fatigue, gastrointestinal symptoms, dyspnea), as well as in the choice of respiratory support, complications (ARDS, septic shock, bacterial infection), and duration of hospitalization (*p* < 0.05). The mortality rate within the cohort was 54%. Comparative analysis between survivors and non-survivors indicated that the latter group was older, had diminished peripheral oxygen saturation and GCS scores, predominantly exhibited fatigue and gastrointestinal symptoms, had a higher prevalence of cardiovascular and rheumatic immune diseases, required more intensive respiratory support (e.g., HFNC, NIV), and had a higher incidence of ARDS, septic shock, and bacterial infections (*p* < 0.05). These findings are delineated in Table 1.

**Table 1:** Baseline demographic and clinical characteristics of patients with Omicron COVID-19 pneumonia.

Variable	All patients (*n* = 409)	In-hospital mortality		*P*-value	disease severity			*P*-value
		Survivors (*n* = 355)	Non survivors (*n* = 54)		Moderate (*n* = 271)	Severe (*n* = 112)	Critical (*n* = 26)	
Age (years)	78(67–86)	77(66–85)	84(76–89)	<0.001	75(65–84)	82(72–87)	82(74–88)	0.023
Male sex *n* (%)	173(42.2)	156(43.9)	17(31.5)	0.084	128(47.2)	40(35.7)	5(19.2)	0.006
**Habits *n* (%)**								
Smoking	39(9.5)	36(10.1)	3(5.6)	0.285	28(10.3)	7(6.3)	4(15.4)	0.268
Alcohol consumption	19(4.6)	15(4.2)	4(7.4)	0.301	12(4.4)	4(3.6)	3(11.5)	0.211
**Vital signs M(IQR)**								
MBP (mmHg)	94(86–103)	94(86–103)	94(81–105)	0.337	94(87–103)	95(85–103)	92(71–106)	0.327
HR (bpm)	83(76–98)	83(76–97)	89(78–102)	0.226	82(76–95)	88(78–102)	93(75–113)	0.009
RR (bpm)	20(19–21)	20(19–20)	20(18–22)	0.123	20(19–20)	20(19–22)	21(18–28)	<0.001
Temp (°C)	36.6(36.3–37.2)	36.6(36.3–37.2)	36.6(36.4–37.2)	0.759	36.5(36.3–37)	36.8(36.4–37.3)	37(36.5–37.6)	0.03
SPO_2_ (%)	96(92–97)	96(93–98)	90(84–95)	<0.001	97(95–98)	92(87–94)	85(67–95)	<0.001
GCS score	15(15–15)	15(15–15)	14(12–15)	<0.001	15(15–15)	15(14–15)	10(9–15)	<0.001
**Comorbidities *n* (%)**								
Hypertension	209(51)	175(49.3)	34(63)	0.061	129(47.6)	62(55.4)	18(69.2)	0.062
Heart disease	61(14.9)	48(13.5)	13(24.1)	0.043	35(12.9)	21(18.8)	5(19.2)	0.282
Diabetes	131(32)	113(31.8)	18(33.3)	0.826	89(32.8)	36(32.1)	6(23.1)	0.595
Chronic lung disease	44(10.7)	39(11)	5(9.3)	0.703	21(7.7)	19(17)	4(15.4)	0.022
Cerebrovascular disease	120(29.3)	99(27.9)	21(38.9)	0.098	75(27.7)	36(32.1)	9(34.6)	0.567
Malignancy	64(15.6)	51(14.4)	13(24.1)	0.067	42(15.5)	18(16.1)	4(15.4)	0.989
Chronic nephropathy	71(17.3)	58(16.3)	13(24.1)	0.162	42(15.5)	27(24.1)	2(7.7)	0.052
Rheumatic immune disease	25(6.1)	16(4.5)	9(16.7)	0.001	10(3.7)	12(10.7)	3(11.5)	0.016
**Symptoms *n* (%)**								
Cough	355(86.6)	307(86.5)	48(88.9)	0.626	229(84.5)	104(92.9)	22(84.6)	0.084
Expectoration	339(82.7)	293(82.5)	46(85.2)	0.630	216(79.7)	101(90.2)	22(84.6)	0.045
Dyspnea	183(44.6)	156(43.9)	27(50)	0.404	106(39.1)	63(56.3)	14(53.8)	0.006
Chest pain	15(3.7)	14(3.9)	1(1.9)	0.446	11(4.1)	4(3.6)	0	0.574
Fatigue	213(52)	176(49.6)	37(68.5)	0.009	122(45)	74(66.1)	17(65.4)	<0.001
Digestive symptoms	267(65.1)	224(63.1)	43(79.6)	0.017	162(59.8)	89(79.5)	16(61.5)	0.001
Respiratory support *n* (%)				<0.001				<0.001
No oxygen	177(43.2)	173(48)	4(7.4)		173(63.8)	4(3.6)	0	
Nasal tube oxygen	55(13.4)	52(14.6)	3(5.6)		53(19.6)	2(1.8)	0	
Mask oxygen	52(12.7)	41(11.5)	11(20.4)		11(4.1)	40(35.7)	1(3.8)	
HFNC	80(19.5)	65(18.3)	15(27.8)		32(11.8)	44(39.3)	4(15.4)	
NIV	28(6.8)	14(3.9)	14(25.9)		1(0.4)	19(17)	8(30.8)	
IV	17(4.1)	10(2.8)	7(13)		1(0.4)	3(2.7)	13(50)	
**Complications *n* (%)**								
ARDS	141(34.4)	97(27.3)	44(81.5)	<0.001	18(6.6)	101(90.2)	22(84.6)	<0.001
Septic shock	18(4.4)	4(1.1)	14(25.9)	<0.001	4(1.5)	9(8)	5(19.2)	<0.001
Bacterial infection	182(44.4)	140(39.4)	42(77.8)	<0.001	84(31)	76(67.9)	22(84.6)	<0.001
LOS M(IQR)	10(7–15)	10(7–15)	11(5–18)	0.752	9(7–13)	13(8–18)	13(8–26)	<0.001

MBP, mean arterial pressure; HR, heart rate; RR, respiratory rate; NIV, noninvasive ventilation; IV, invasive ventilation; HFNC, high-flow nasal cannula; Temp, temperature; SPO_2_, arterial partial pressure of oxygen; GCS, Glasgow coma scale; ARDS, acute respiratory distress syndrome; LOS, length of stay; M(IQR), median (interquartile range).

### 3.2 Laboratory findings and scoring systems

The severity of the disease correlated positively with elevated levels of WBC, neutrophil-to-lymphocyte ratio (NLR), CRP, PCT, interleukin-6, BUN, creatinine, aspartate aminotransferase (AST), lactate dehydrogenase (LDH), glucose, creatine kinase (CK), creatine kinase-MB (CK-MB), troponin I, N-terminal pro b-type natriuretic peptide (NT-proBNP), D-dimer, fibrinogen, and lactate. Moreover, the CURB-65, PSI score, and MuLBSTA scores, alongside mortality rates, escalated with disease severity. In the non-survival group, markers such as NLR, CRP, PCT, interleukin-6, BUN, creatinine, AST, LDH, glucose, CK-MB, troponin, NT-proBNP, D-dimer, fibrinogen, and lactate were significantly higher, whereas lymphocyte counts, serum albumin levels, and the oxygenation index were lower compared to survivors. The non-survival group also recorded higher CURB-65, PSI, and MuLBSTA scores, indicative of more severe clinical categorization (*p* < 0.05). These results are summarized in Table 2.

**Table 2:** Laboratory results and various illness prediction scoring systems of the groups.

Variable	All patients (*n* = 409)	In-hospital mortality		*P*-value	Disease Severity			*P*-value
		Survivors (*n* = 355)	Non survivors (*n* = 54)		Moderate (*n* = 271)	Severe (*n* = 112)	Critical (*n* = 26)	
**Laboratory results M(IQR)**								
WBC (*10^9^/L)	6.17(4.60–9.29)	6.13(4.57–8.84)	6.47(4.77–12.66)	0.092	5.94(4.57–8.53)	6.47(4.47–9.8)	10.88(6.33–15.23)	<0.001
Neutrophil (*10^9^/L)	4.65(3.09–7.53)	4.56(2.95–6.92)	5.83(3.53–10.90)	0.013	4.3(2.8–6.34)	5.18(3.46–8.10)	9.42(4.76–13.24)	<0.001
Lymphocyte (*10^9^/L)	0.89(0.6–1.37)	0.93(0.62–1.41)	0.65(0.39–1.16)	<0.001	0.98(0.65–1.54)	0.80(0.47–1.08)	0.82(0.45–1.28)	<0.001
NLR	5.01(2.7–9.68)	4.73(2.52–8.68)	9.13(4.26–23.04)	<0.001	4.18(2.24–7.67)	6.51(4.16–14.46)	7.97(4.56–23.53)	<0.001
Hemoglobin (g/L)	118(105–132)	119(107–132)	114(99–132)	0.212	120(108–133)	116.5(101.25–131)	112(102.75–132.25)	0.276
Platelet (*10^9^/L)	173.00(124.50–224.00)	174.00(134.00–224.00)	143(105–217)	0.076	172(129–224)	171(122.5–220)	188.5(107.75–255.75)	0.731
CRP (mg/L)	29.20(9.26–84.47)	23.59(7.02–74.09)	89.34(42.88–142.65)	<0.001	18.85(5.49–59.28)	71.65(22.18–118.66)	82.18(50.27–138.74)	<0.001
PCT (ng/ml)	0.21(0.07–0.56)	0.18(0.06–0.5)	0.51(0.20–2.11)	<0.001	0.12(0.05–0.45)	0.34(0.1–0.86)	0.85(0.44–2.57)	<0.001
Interleukin-6 (pg/mL)	10.40(4.89–21.50)	10.09(4.50–19.09)	23.18(11.90–84.86)	<0.001	9.06(4.44–15.40)	13.21(5.5–51.55)	31.35(12.13–99.12)	<0.001
Blood urea nitrogen (mmol/L)	6.64(4.79–11.17)	6.18(4.58–10.20)	11.25(7.23–17.12)	<0.001	6.03(4.42–9.54)	8.4(5.57–13.23)	10.32(7.31–16.84)	<0.001
Creatinine (μmol/L)	79.00(64.00–114.20)	77.00(63.00–109.02)	99.00(73.84–187.30)	0.001	75(60.69–110)	92.88(68.28–141.53)	87.85(73.63–118.37)	0.006
Serum albumin (g/L)	35.70(32.10–39.50)	36.40(33.00–39.80)	30.79(28.30–33.95)	<0.001	36.9(33.59–40.4)	33.1(29.85–36.76)	32.49(29.95–36.63)	<0.001
ALT (U/L)	19.20(13.35–30.90)	19.20(13.70–30.00)	19.45(12.18–32.75)	0.739	18.6(13–30)	20.4(14.6–29)	22(12.6–44.75)	0.359
AST (U/L)	28.00(20.20–41.00)	26.70(20.10–39.82)	32.90(23.46–56.58)	0.019	24.3(19.1–37.2)	33.75(24.03–51.11)	43.6(26.36–93.07)	<0.001
Serum LDH (U/L)	231.02(190.20–279.45)	226.00(185.96–265.00)	325.88(230.58–490.74)	<0.001	217(180.97–250)	267.5(227.5–351.75)	313.87(215.43–460.36)	<0.001
Glucose (mmol/L)	6.50(5.14–8.34)	6.18(5.04–8.07)	7.93(6.67–11.84)	<0.001	6.16(4.99–8.14)	6.76(5.5–8.34)	7.06(5.83–10.47)	0.025
CK (U/L)	76.00(40.83–160.85)	75.00(41.00–144.00)	89.50(36.93–220.43)	0.246	64(39–125)	103.925(50.15–196.9075)	175.43(47.98–284.95)	<0.001
CK-MB (U/L)	12.20(8.95–18.25)	11.99(9.00–16.80)	15.87(7.9–27.83)	0.01	11.8(8.9–16.3)	13.46(9.01–21.05)	15.46(7.76–26.31)	0.04
Troponin I (ng/mL)	0.02(0.01–0.03)	0.02(0.01–0.03)	0.04(0.02–0.24)	<0.001	0.02(0.01–0.03)	0.02(0.01–0.04)	0.04(0.02–0.13)	<0.001
NT-proBNP (pg/mL)	551.2(133.55–1219.33)	410.85(122.10–1006.20)	1707.75(793.95–9194.37)	<0.001	330.6(117.63–992.75)	890.3(338.86–2417.59)	1275.77(796.32–8273.24)	<0.001
Fibrinogen (g/L)	3.96(3.12–5.33)	3.93(3.10–5.07)	5.05(3.36–6.38)	0.003	3.85(3.01–4.67)	4.88(3.42–6.10)	4.00(3.21–5.54)	<0.001
D-dimer (mg/L)	0.75(0.44–1.65)	0.62(0.41–1.41)	1.72(0.98–3.24)	<0.001	0.55(0.4–1.23)	1.09(0.55–2.27)	2.01(1.03–6.48)	<0.001
Potassium (mmol/L)	4.03(3.62–4.43)	4.02(3.62–4.41)	4.16(3.53–4.71)	0.269	4.01(3.63–4.43)	4.08(3.59–4.47)	4.15(3.42–4.49)	0.809
Blood sodium (mmol/L)	136.46(132.76–139.19)	136.55(133.14–139.08)	135.26(129.41–139.93)	0.398	136.76(133.23–139.14)	136.08(131.485–139.31)	134.13(127.44–140.32)	0.508
Lactate acid (mmol/l)	1.29(1.09–1.65)	1.23(1.09–1.56)	1.71(1.12–2.09)	<0.001	1.2(1.09–1.47)	1.36(1.12–1.89)	1.92(1.07–2.79)	<0.001
PaO_2_/FIO_2_(P/F)	318.62(258.19–385.95)	324.83 (278.97–390.95)	182.62(121.83–269.40)	<0.001	346.2(317.59–420.95)	241.03(172.16–272.79)	169.72(96.15–206.59)	<0.001
**Critically illness prediction scores, M(IQR)**								
CURB-65 score	1(1–2)	1(1–2)	2(2–3)	<0.001	1(1–2)	2(1–2)	3(2–4)	<0.001
PSI score	101(77–128)	95(74–121)	139(124–169)	<0.001	92(70–112)	125(93–142)	138(119–174)	<0.001
MulBSTA score	11(7–13)	9(7–13)	15(11–17)	<0.001	9(7–13)	13(11–17)	13(13–17)	<0.001
Clinical classification *n* (%)				<0.001				
Moderate	271(66.1)	262(73.8)	9(16.7)			-	-	-
Severe	112(27.3)	82(23.1)	30(55.6)			-	-	-
Critical	26(6.3)	11(3.1)	15(27.8)			-	-	-
Death	-	-	-		9(3.3)	30(26.8)	15(57.7)	<0.001

WBC, white blood cell; NLR, neutrophil-to-lymphocyte ratio; CRP, C-reactive protein; PCT, procalcitonin; ALT, alanine aminotransferase; AST, aspartate aminotransferase; LDH, Lactate dehydrogenase; CK, creatine kinase; CK-MB, creatine kinase-MB; PaO_2_/FIO_2_(P/F), oxygenation index; M(IQR), median (interquartile range).

### 3.3 Independent risk factors for disease severity and mortality

Upon merging the severe and critical categories into a “critical” group and classifying the moderate cases as “ordinary,” a multivariate logistic regression analysis was conducted using select variables (age, sex, chronic pulmonary diseases, rheumatic immune disorders, sputum production, fatigue, gastrointestinal symptoms, dyspnea, body temperature, heart rate, respiratory rate, oxygenation index, GCS score, WBC count, neutrophil count, lymphocyte count, NLR, CRP, PCT, interleukin-6, BUN, creatinine, serum albumin, AST, LDH, glucose, CK, CK-MB, troponin I, NT-proBNP, fibrinogen, D-dimer, lactate, ARDS, septic shock, bacterial infection) as predictors and disease severity as the outcome. The analysis identified sputum production, gastrointestinal symptoms, GCS score, creatinine, LDH, PCT, and ARDS as independent predictors of disease severity (*p* < 0.05), detailed in Table 3.

**Table 3:** Multivariate regression analysis of severity-related variables in patients with Omicron COVID-19 pneumonia.

Variable	*β*	SE	Wald χ^2^	OR	95%CI	*P*-value
Expectoration	1.577	0.577	7.455	4.838	(1.560,15.002)	0.006
Digestive symptoms	1.155	0.486	5.644	3.175	(1.224,8.235)	0.018
GCS	−0.475	0.157	9.181	0.622	(0.457,0.845)	0.002
ARDS	5.491	0.520	111.528	242.437	(87.506,671.675)	<0.001
PCT	−0.029	0.012	5.36	0.972	(0.948,0.996)	0.021
Creatinine	0.003	0.001	13.13	1.003	(1.001,1.005)	<0.001
LDH	0.006	0.002	8.641	1.006	(1.002,1.009)	0.003

GCS, Glasgow coma scale; ARDS, acute respiratory distress syndrome; PCT, procalcitonin; LDH, lactate dehydrogenase.

Further, a logistic regression analysis considering the aforementioned significant variables (age, rheumatic immune disorders, heart disease, fatigue, gastrointestinal symptoms, oxygenation index, GCS score, neutrophil count, lymphocyte count, NLR, CRP, PCT, interleukin-6, BUN, creatinine, serum albumin, AST, LDH, glucose, CK-MB, troponin I, NT-proBNP, fibrinogen, D-dimer, lactate, ARDS, septic shock, bacterial co-infection) against mortality as the outcome revealed age, oxygenation index, LDH and glucose levels, and septic shock as independent mortality predictors in COVID-19 pneumonia patients (*p* < 0.05), as shown in Table 4.

**Table 4:** Multivariate regression analysis of mortality in patients with Omicron COVID-19 pneumonia.

Variable	*β*	SE	Wald χ^2^	OR	95%CI	*P*-value
Age	0.068	0.021	10.773	1.070	(1.028,1.115)	0.001
PaO_2_/FIO_2_	−0.011	0.002	28.312	0.989	(0.985,0.993)	<0.001
Septic shock	3.005	0.664	20.480	20.177	(5.492,74.128)	<0.001
LDH	0.005	0.001	12.472	1.005	(1.002,1.007)	<0.001
Glucose	0.097	0.035	7.822	1.102	(1.029,1.179)	0.005

PaO2/FIO2, oxygenation index; LDH, lactate dehydrogenase.

### 3.4 Predictive efficacy of scoring systems for disease severity

**Figure 2:**
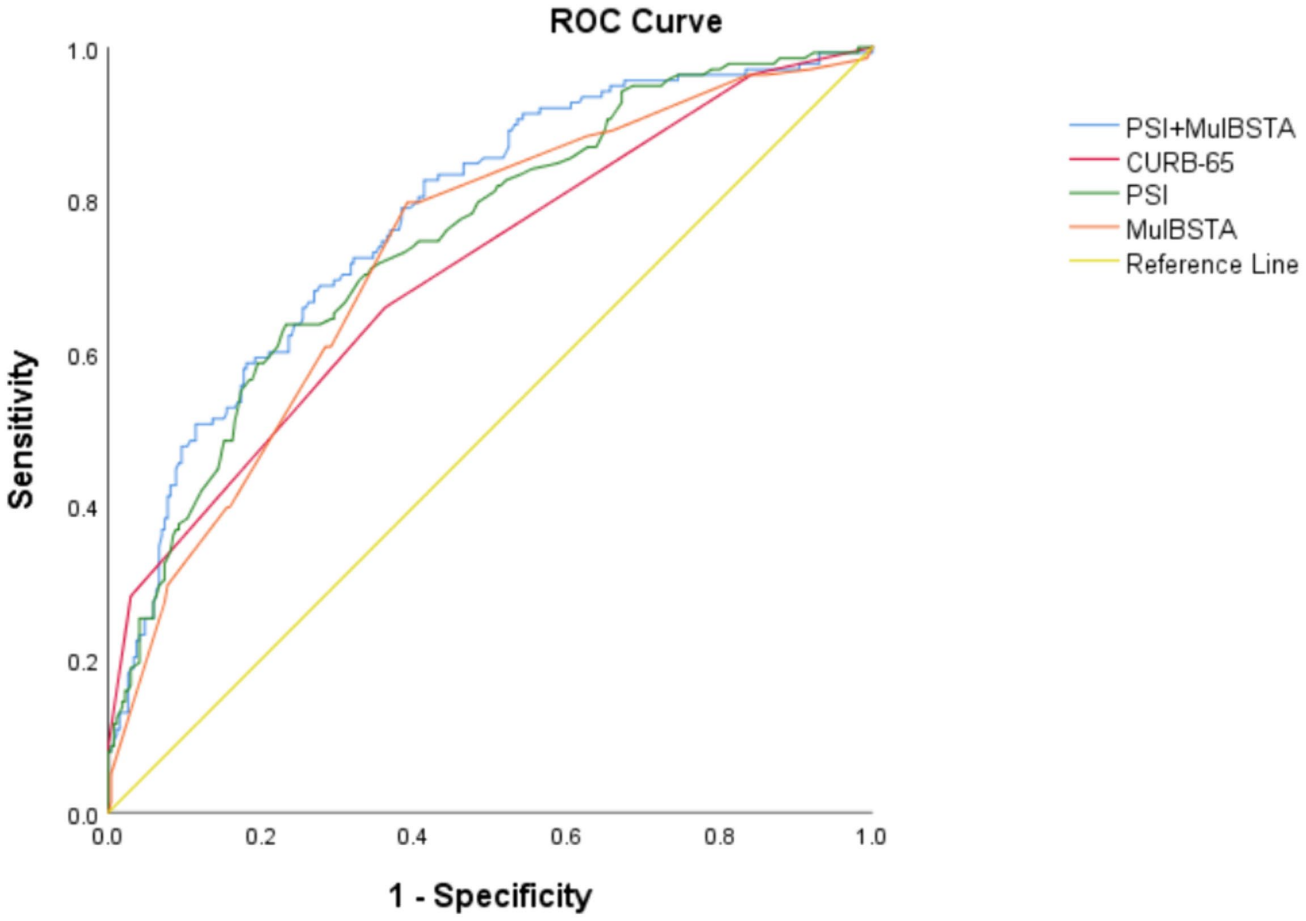
ROC curve of various indexes predicting severity of patients with Omicron COVID-19 pneumonia.

AUROC curves for the CURB-65, PSI, and MuLBSTA scores in predicting critical illness were 0.707, 0.75, and 0.728, respectively. At defined thresholds (CURB-65 ≥ 1.5, PSI ≥ 115.5, MuLBSTA ≥10.5), the sensitivity for critical illness prediction was 65.9, 63.8, and 79.7%, respectively, with specificities of 63.8, 76.8, and 60.9%. The combined PSI and MuLBSTA scores improved the AUROC, sensitivity, and specificity to 0.777, 82.6, and 58.7%, respectively. These findings are elaborated in Table 5 and illustrated in Figure 2. Excluding the combined PSI and MuLBSTA scores, the PSI exhibited superior AUROC, significantly outperforming the CURB-65 score, as represented in Tables 5, 6 and Figure 2.

**Table 5:** The predictive value of CURB-65, PSI, and MulBSTA on the risk of severity in patients with Omicron COVID-19 pneumonia.

Assessment criteria	AUC	Sensibility	Specificity	PPV	NPV
CURB-65 ≥ 1.5	0.707(0.653,0.761)	0.659(0.573,0.737)	0.638(0.578,0.695)	0.481(0.409,0.555)	0.786(0.725,0.837)
PSI ≥ 115.5	0.750(0.700,0.799)	0.638(0.551,0.717)	0.768(0.712,0.816)	0.583(0.500,0.662)	0.806(0.752,0.852)
MulBSTA≥10.5	0.728(0.677,0.780)	0.797(0.718,0.859)	0.609(0.548,0.667)	0.509(0.440,0.577)	0.855(0.795,0.900)
PSI+ MulBSTA	0.777(0.730,0.824)	0.826(0.750,0.883)	0.587(0.525,0.646)	0.504(0.438,0.571)	0.869(0.809,0.913)

**Table 6:** Comparison of the AUROC curve between the several scoring models.

	*Z*	CI	*p*-value
**Severity of disease**			
PSI+ MulBSTA vs. CURB65	3.343	(0.029,0.111)	0.001
PSI+ MulBSTA vs. PSI	2.470	(0.006,0.049)	0.014
PSI+ MulBSTA vs. MulBSTA	2.670	(0.013,0.085)	0.008
CURB65 vs. PSI	−2.056	(−0.083,-0.002)	0.040
CURB65 vs. MulBSTA	−0.729	(−0.078,0.036)	0.466
PSI vs. MulBSTA	0.774	(−0.033,0.075)	0.439
**Death**			
Clinical classification vs. CURB-65	0.533	(−0.061,0.106)	0.594
Clinical classification vs. PSI	−1.092	(−0.115,0.033)	0.275
Clinical classification vs. MulBSTA	1.577	(−0.018,0.164)	0.115
Clinical classification vs. Clinical classification +PSI	−4.197	(−0.139, −0.051)	<0.001
CURB65 vs. PSI	−2.243	(−0.119, −0.008)	0.025
CURB65 vs. MulBSTA	1.240	(−0.029, 0.130)	0.215
CURB65 vs. Clinical classification +PSI	−4.000	(−0.175, −0.060)	<0.001
PSI vs. MulBSTA	3.240	(0.045, 0.183)	0.001
PSI vs. Clinical classification +PSI	−2.888	(−0.090, −0.017)	0.004
MulBSTA vs. Clinical classification +PSI	−4.787	(−0.237, −0.099)	<0.001

### 3.5 Predictive efficacy of scoring systems for mortality

**Figure 3:**
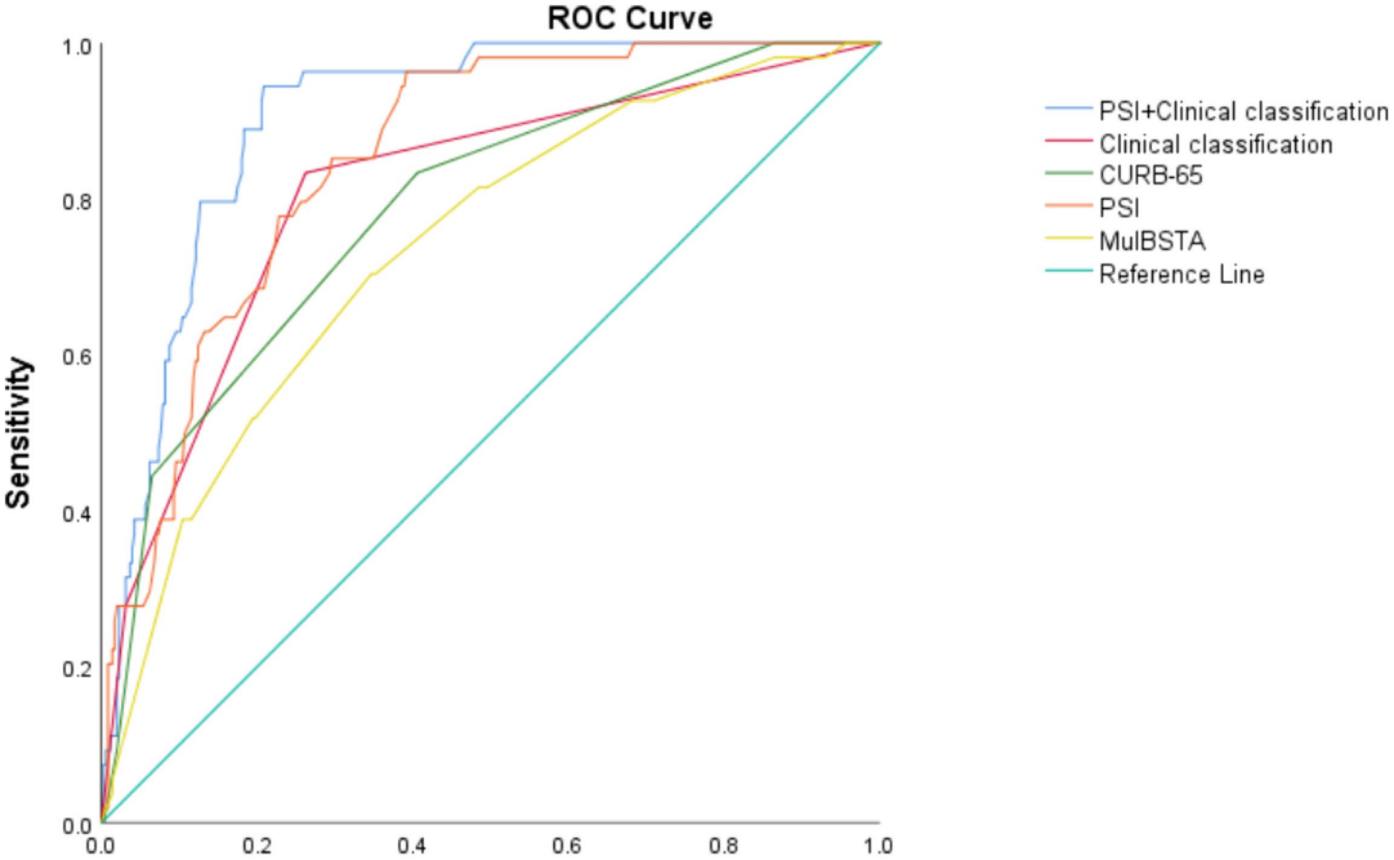
ROC curve of various indexes predicting death of patients with Omicron COVID-19 pneumonia.

The predictive accuracy for mortality, as measured by AUROC, for the CURB-65, PSI, MuLBSTA scores, and clinical classification were 0.787, 0.85, 0.736, and 0.809, respectively. Thresholds set at CURB-65 ≥ 1.5, PSI ≥ 102.5, and MuLBSTA ≥ 12.5 yielded mortality prediction sensitivities of 83.8%, 96.3%, and 70.4%, with specificities of 59.4%, 60.8%, and 65.4%. Severe or higher clinical classification demonstrated an AUROC of 0.809, with sensitivity and specificity for mortality prediction at 83.3% and 73.8%. Combining clinical classification with the PSI score enhanced the AUROC to 0.904, with sensitivity and specificity rates of 94.4 and 79.2%, respectively. These outcomes are detailed in Table 7 and Figure 3. Excluding the combined PSI and clinical classification, the PSI score alone had the highest AUROC, significantly surpassing both CURB-65 and MuLBSTA scores, as indicated in Tables 6, 7 and Figure 3.

**Table 7:** The predictive value of CURB-65, PSI, MulBSTA and clinical classification of treatment regimens on the risk of death in patients with Omicron COVID-19 pneumonia.

Assessment criteria	AUC	Sensitivity	Specificity	PPV	NPV
CURB-65 ≥ 1.5	0.787(0.722,0.851)	0.833(0.702,0.916)	0.594(0.541,0.646)	0.238(0.181,0.306)	0.959(0.921,0.980)
PSI ≥ 102.5	0.850(0.805,0.896)	0.963(0.862,0.994)	0.608(0.555,0.659)	0.272(0.212,0.342)	0.990(0.964,0.998)
MulBSTA ≥ 12.5	0.736(0.667,0.806)	0.704(0.562,0.816)	0.654(0.601,0.702)	0.236(0.174,0.311)	0.935(0.895,0.961)
Clinical classification	0.809(0.745,0.874)	0.833(0.702,0.916)	0.738(0.688,0.782)	0.326(0.250,0.412)	0.967(0.936,0.984)
Clinical classification +PSI	0.904(0.871,0.937)	0.944(0.837,0.986)	0.792(0.745,0.832)	0.408(0.322,0.500)	0.989(0.967,0.997)

## 4 Discussion

COVID-19 is a grave respiratory infectious disease noted for its significant mortality rate. Studies have shown that 17% of patients with COVID-19 who develop ARDS deteriorate rapidly and succumb, particularly in the initial outbreak phase in Wuhan, Hubei Province (8, 9). This highlights the critical need for early identification of patients at high risk and the implementation of prompt and effective symptomatic support to enhance prognosis. The stratification of COVID-19 patient severity and prognostic assessment are vital yet challenging domains in clinical research and practice.

In this study, 409 patients infected with the Omicron variant of COVID-19 pneumonia were analyzed, revealing an in-hospital mortality rate of 13.2%. By contrast, an Italian study with 1,715 patients reported ICU and hospital mortality rates of 48.8 and 53.4%, respectively (10), and another study of 344 COVID-19 patients in ICUs showed an ICU mortality rate of 58.3% (11). A cohort of 138 confirmed cases in Wuhan reported a mortality rate of 4.3% (12). Despite the high transmissibility but lower pathogenicity of the Omicron variant, it can still lead to severe outcomes in individuals with comorbidities (13), which may explain the higher mortality rate in our cohort, likely due to the inclusion of elderly patients with underlying diseases. The pathogenicity of Omicron was lower than that of Delta, and its transmission ability was 3.3 times higher than that of Delta mutant (14). The Beta mutant has a higher transmission rate and mortality than the Alpha mutant, the Delta variant is more contagious than the original strain and other variants, and the risk of hospitalization is found to be twice as high as that of the alpha variant. A recent study shows that hospitalized patients infected with Delta are more ill and have higher hospital mortality (15). Systematic reviews suggest that the Omicron variant’s severity is less than that of previous waves (16), although some studies indicate that Omicron-infected patients may exhibit severity comparable to other variants. Notably, infection severity among hospitalized patients during the Omicron wave may be reduced, yet the risk of severe illness persists, with the virus more likely affecting older males with comorbidities, potentially leading to severe or fatal respiratory diseases like ARDS (9, 17). Previous studies have identified age, comorbidities, and ARDS as independent mortality predictors, with a 61.5% mortality rate in critically ill patients, and the oxygenation index being associated with disease severity and prognosis (12, 18, 19).

Studies have shown that early inhibition of virus replication can significantly improve the prognosis, patients who received Paxlovid presented a more rapid virus suppression at the initial stage of hospitalization and an earlier RT-PCR negative conversion than those who received Azvudine (20). In a retrospective cohort study, Azvudine does not reduce the rate of all-cause death (21), which is more suitable for man under the age of 65 years (22). Besides, a study showed that combining l-Arginine with vitamin C improves long-COVID symptoms by reducing oxidative stress. This study included the treatment plan of patients as individualized treatment, which mainly included oxygen therapy, hormone therapy, anti-infection, antiviral therapy and so on. 181patients received antiviral therapy in our study, 72 patients received Paxlovid, 92patients received Azvudine, 1patient received Molnupiravir, and 16 patients received sequential Azvudine and Paxlovid.

This study included hospitalized COVID-19 pneumonia patients, with a median patient age of 75 years, identified as an independent risk factor for poor prognosis. Over 80% of the patients had underlying diseases, with about 32% having two or more comorbidities. The most prevalent underlying diseases were hypertension (49.3%) and diabetes mellitus (31.8%). The proportion of non-survivors with heart disease and rheumatic immune disease was significantly higher than that of survivors. Moreover, the severity of the condition was linked with an increased likelihood of high blood pressure, chronic lung disease, and rheumatic immune disease, indicating that cardiovascular disease and immunosuppression may exacerbate the disease and elevate mortality risk. However, hypertension was not identified as an independent factor for mortality in the multivariate analysis, possibly due to the inclusion of generally older patients and other laboratory indicators that might diminish the predictive value of complications.

The incidence of ARDS in the mortality and critically ill groups was significantly higher than in their respective control groups, establishing ARDS as an independent risk factor for critical illness. In line with prior studies (12, 18, 23, 24), non-survivors and critically ill patients in this study had a lower oxygenation index, indicating that critically ill patients often require more advanced respiratory support. The oxygenation index also emerged as an independent risk factor for mortality, highlighting hypoxia’s role in disease prognosis and severity.

Most patients in this study presented symptoms such as cough, expectoration, and digestive symptoms, consistent with previous findings (12). The main symptoms in the critically ill group were expectoration, fatigue, digestive symptoms, and dyspnea, while fatigue and digestive symptoms were more prevalent in the mortality group. Expectoration and digestive symptoms were identified as independent risk factors for critical conditions, suggesting that patients with a poorer general condition are more susceptible to severe illness. The study also underscored the high risk of septic shock due to concomitant bacterial infection (9), with a higher proportion of patients in the mortality and critically ill groups experiencing bacterial infections. Septic shock was pinpointed as an independent risk factor for death, emphasizing the importance of timely identification and treatment of bacterial infections in these patients. The impact of consciousness changes on patient prognosis has been less explored; however, Xiong et al. (25) found a significant association between lower GCS scores and mortality. Another study (26) demonstrated that disturbances in consciousness are independent predictors of critical disease development. Despite the dynamic nature of consciousness and various potential confounding factors, this study found that GCS scores within 24 h of admission served as independent risk factors for critical illness, indicating their prognostic value for disease progression.

The role of laboratory examinations in predicting disease severity and mortality risk has been a focal point of recent research, bearing significant clinical implications for establishing and comparing scoring systems. Ruan et al. (19) observed notable differences in various parameters between survival and non-survival groups, including white blood cell count, lymphocyte count, platelet count, serum albumin, total bilirubin, BUN, creatinine, myoglobin, Troponin I, CRP and Interleukin-6. Additionally, Mo et al. (27) found that refractory COVID-19 patients exhibited higher levels of neutrophils, aspartate aminotransferase, lactate dehydrogenase, and CRP, along with lower levels of platelets and serum albumin. Refractory patients were also more likely to require mechanical ventilation. A meta-analysis revealed that changes in WBC, neutrophil, and lymphocyte counts were associated with critical condition and mortality risk in COVID-19 patients (28). Furthermore, a retrospective study by Bernal-Monterde et al. (29) showed that elevated levels of gamma-glutamyl transpeptidase and alkaline phosphatase, along with decreased albumin levels, were linked to an increased mortality risk in COVID-19 patients. A literature review outlined the main risk factors for severe clinical progression and adverse outcomes in COVID-19 patients, which included coagulation disorders, leukocytosis, lymphopenia, decreased eosinophils, and elevated levels of aspartate aminotransferase, BUN creatine kinase, troponin I, CRP, PCT, and Interleukin-6. In this study, lymphocyte counts and serum albumin levels were lower in the critically ill and mortality groups (30). Conversely, NLR and CRP, PCT, Interleukin-6, BUN, creatinine, AST, LDH, glucose, CK-MB, troponin I, NT-ProBNP, fibrinogen, D-dimer, and lactate levels were higher. Previous studies have suggested that a significant decrease in the total lymphocyte count indicates that the coronavirus depletes immune cells and hampers cellular immune function (31). T lymphocyte damage may be a key factor in patient deterioration (11), and the extent of the increase in neutrophil count may reflect the intensity of the inflammatory response in COVID-19 patients, linked to the cytokine storm induced by virus invasion (12). Additionally, coagulation activation may be associated with persistent inflammation, and the degree of lymphocyte count reduction also indicates the level of immune system damage caused by viral infection (32). Thus, NLR can serve as a useful indicator to reflect the imbalance of inflammation and immune response in COVID-19 patients (31).

The levels of creatinine and BUN were higher in COVID-19 patients in the critically ill and mortality groups, indicating that the novel coronavirus significantly affects the human kidney. Acute renal injury may result from the direct effects of viruses, hypoxia, and shock (12). A study involving 701 patients showed that an increase in creatinine level upon admission correlated with disease severity (33), consistent with the findings of this study. Another study examining refractory patients reported significantly elevated LDH and CRP levels. LDH is a predictor of inflammation in various lung diseases, such as obstructive disease, interstitial lung disease, and severe pneumonia (27). CRP, a widely used biochemical marker of inflammation, reflects acute and severe systemic inflammatory responses resulting from viral infection. A recent study indicated that COVID-19 patients treated in the ICU exhibited higher levels of LDH and CRP than patients who did not receive ICU treatment (12), suggesting that CRP and LDH levels are primarily associated with a poor prognosis. In this study, LDH emerged as an independent risk factor for critical illness and mortality, whereas CRP level did not. It is noteworthy that some studies have demonstrated that CRP cannot directly reflect cytotoxicity to the same extent as LDH, indicating that it may not be as effective as LDH in evaluating the severity and prognosis of COVID-19 pneumonia (34). Furthermore, several studies have shown a correlation between high glucose levels and poor prognosis (35), consistent with the results of the present study. The increase in glucose levels may be attributed to stress caused by inflammation and damage to islet cells.

Except for the above laboratory indicators, SARS-CoV-2 nucleocapsid antigen and Krebs Von den Lungen-6 are worthy of attention. A large, multicenter cohort confirms that plasma SARS-CoV-2 nucleocapsid antigen is highly associated with both baseline severity of lung illness and clinically patient outcomes, it is suggested that viral replication may play a potential role in the pathogenesis of SARS-CoV-2 in hospitalized patients (36); Krebs Von den Lungen-6 is a glycoprotein expressed mainly from type II alveolar cells with pro-fibrotic properties, which mainly reflects the degree of interstitial lung impairment (37), and it could be used to predict the risk of oro-tracheal intubation or death among hospitalized severe or critical COVID-19 patients (38); look forward to further verification by prospective studies.

The PSI and CURB-65 scores have been subjected to retrospective analysis of large samples in community-acquired pneumonia (CAP) and have proven effective in accurately stratifying CAP patients and assisting clinicians in making medical decisions (39, 40). The MuLBSTA score, developed by Chinese scholars, has emerged as an early warning model for predicting viral pneumonia mortality in recent years (5), assessing the 90-day risk of death in patients with viral pneumonia and demonstrating a sensitivity of 77.6% and a specificity of 77.8%, outperforming the CURB-65 score (AUROC = 0.773 vs. 0.717, *p* < 0.001). Moreover, the MuLBSTA score accurately categorized hospitalized patients with viral pneumonia based on related risk levels, providing guidance for further clinical decision-making. Satici et al. (41) also reported the superiority of the PSI score over the CURB-65 score in a series of cases of Turkey. Similarly, one study of Spain found that the PSI score is valuable in predicting mortality risk of COVID-19 CAP (42), which is consistent with our findings. In our study, the PSI score outperformed the CURB-65 score in predicting disease severity. When combined with the MuLBSTA score, the PSI score demonstrated the highest effectiveness in predicting disease severity, with an AUROC of 0.777, sensitivity of 82.6%, and specificity of 58.7%. When used to predict the risk of mortality, the PSI score exhibited higher efficiency than the MuLBSTA and CURB-65 scores, with AUROCs of 0.85, 0.787, and 0.736, respectively. The sensitivities for predicting mortality were 96.3%, 83.3%, and 70.4%, while the specificities were 60.8%, 59.4%, and 65.4%, respectively. A higher risk of mortality was observed when the PSI score was ≥102.5, the CURB-65 score was ≥1.5, and the MuLBSTA score was ≥12.5, respectively. In this study, we aimed to use the clinical classification of moderate, severe, and critical factors to predict patient mortality. The results demonstrated that, for severe and critical cases, the risk of mortality was higher, with an AUC of 0.809. The sensitivity and specificity of the mortality prediction were 83.3% and 73.8%, respectively. Furthermore, we sought to assess mortality risk by combining the PSI score and clinical classification. Our findings revealed that the combination of these two models yielded higher efficacy than either model individually, with an AUROC of 0.904. The sensitivity and specificity of the mortality prediction were 94.4% and 79.2%, respectively. Moreover, when considering the prediction of both outcomes, the PSI score exhibited the highest AUROC. Consequently, the PSI score has superior value in predicting disease severity and mortality risk, likely due to its multiple evaluation parameters (age, oxygenation, vital signs and complications) and comprehensive assessment capabilities. It is worth noting that while the PSI score lacks specific viral infection indices in its parameters, some studies have shown that the risk of death from both viral and non-viral CAP increases with higher PSI grades. This suggests that age, vital signs, and complications may have a more substantial impact on prognosis, which aligns with the findings of our study (43).

Previous studies have demonstrated that the MuLBSTA score performs well in predicting mortality and risk stratification (44), the MuLBSTA score outperformed the PSI score in predicting the need for ICU admission. The PSI score places significant emphasis on the influence of age and comorbidities on prediction, potentially leading to an underestimation of risk for certain patients, particularly younger individuals without comorbidities. Additionally, the CURB-65 score focuses on vital sign monitoring to distinguish patients at a high risk of mortality due to severe CAP, offering a simple and practical approach. However, it overlooked the impact of comorbidities on the disease.

This was a single-center retrospective study with certain limitations. The general condition of the patients included in the study was poor as they were generally older and had multiple underlying diseases. Additionally, the severity of the disease was high, leading to a selection bias in the patient population. It is important to note that we did not follow-up on discharged patients, which potentially impacted our findings. Moreover, owing to the lack of survival time data, the mortality rate may have been underestimated.

Furthermore, as this was a retrospective study, there was an inherent information bias. Additionally, the results of this study have not been validated in different hospitals, potentially affecting the generalizability of the findings. Therefore, further validation through a multicenter prospective study is necessary.

Another limitation of our study is that it did not include a large number of asymptomatic and mild patients, as well as those treated at home or outpatient clinics. Hence, our cohort may represent a more severe outcome for Omicron COVID-19 pneumonia.

It is essential to acknowledge that the Omicron COVID-19 pneumonia is a novel respiratory virus with constantly evolving strains. As our understanding of its basic and clinical aspects is still being explored and updated, a comprehensive assessment of the risk of disease severity and mortality requires large-scale clinical studies for confirmation.

## 5 Conclusion

During the Omicron variant outbreak, the clinical presentations of patients exhibited subtle distinctions compared to earlier phases of the pandemic. Notably, factors such as hypoxia, severe infections, and elevated LDH levels emerged as more predictive of critical illness and mortality. Regarding the assessment of disease severity, the PSI demonstrated significant utility, particularly when used in conjunction with the MuLBSTA score, enhancing its predictive accuracy. This study revealed that clinical categorization, along with PSI and CURB-65 scores, were effective in forecasting mortality risk. Among these, the PSI score showed superior predictive performance. Its predictive capability was further amplified when combined with clinical classification, offering the highest prognostic value. In light of these findings, it is advisable to employ the PSI score for evaluating the severity and mortality risk associated with Omicron COVID-19 pneumonia. Where feasible, integrating the PSI score with other assessments is recommended to achieve a more comprehensive risk evaluation.

## Data availability statement

The raw data supporting the conclusions of this article will be made available by the authors, without undue reservation.

## Ethics statement

The studies involving humans were approved by the Clinical Research Ethics Committee of the Third Affiliated Hospital of Anhui Medical University granted ethical. The studies were conducted in accordance with the local legislation and institutional requirements. The participants provided their written informed consent to participate in this study. Written informed consent was obtained from the individual(s) for the publication of any potentially identifiable images or data included in this article.

## Author contributions

RN: Data curation, Formal analysis, Investigation, Methodology, Software, Supervision, Validation, Writing – original draft, Writing – review & editing, Conceptualization. MZ: Conceptualization, Funding acquisition, Investigation, Project administration, Resources, Software, Supervision, Validation, Writing – original draft, Writing – review & editing, Visualization. MX: Conceptualization, Formal analysis, Investigation, Validation, Writing – original draft, Software. ZD: Formal analysis, Methodology, Supervision, Visualization, Writing – review & editing.

## Glossary

COVID-19: Coronavirus Disease 2019
WHO: World Health Organization
Diagnosis and Treatment Plan: *Diagnosis and Treatment Plan for Novel Coronavirus Infection -Tenth edition for trial implementation*
Clinical classification: Clinical classification of Diagnosis and Treatment Plan
MBP: Mean arterial pressure
HR: Heart rate
RR: Respiratory rate
NIV: Noninvasive ventilation
IV: Invasive ventilation
HFNC: High-flow nasal cannula
Temp: Temperature
SPO_2_: Arterial partial pressure of oxygen
GCS: Glasgow coma scale
ARDS: Acute respiratory distress syndrome
LOS: Length of stay
WBC: White blood cell
NLR: Neutrophil-to-lymphocyte ratio
CRP: C-Reactive protein
PCT: Procalcitonin
ALT: Alanine aminotransferase
AST: Aspartate aminotransferase
LDH: Lactate dehydrogenase
CK: Creatine kinase
CK-MB: Creatine kinase-MB
PaO_2_/FIO_2_(P/F): Oxygenation index
M(IQR): median (interquartile range)
CI: Confidence interval
